# Copy Number Variation of *CCL3-*like Genes Affects Rate of Progression to Simian-AIDS in Rhesus Macaques (*Macaca mulatta*)

**DOI:** 10.1371/journal.pgen.1000346

**Published:** 2009-01-23

**Authors:** Jeremiah D. Degenhardt, Paola de Candia, Adrien Chabot, Stuart Schwartz, Les Henderson, Binhua Ling, Meredith Hunter, Zhaoshi Jiang, Robert E. Palermo, Michael Katze, Evan E. Eichler, Mario Ventura, Jeffrey Rogers, Preston Marx, Yoav Gilad, Carlos D. Bustamante

**Affiliations:** 1 Biological Statistics and Computational Biology, Cornell University, Ithaca, New York, United States of America; 2 Department of Human Genetics, University of Chicago, Chicago, Illinois, United States of America; 3 Tulane National Primate Research Center, Covington, Louisiana, United States of America; 4 Department of Genome Sciences, University of Washington, Seattle, Washington, United States of America; 5 Department of Microbiology, University of Washington, Seattle, Washington, United States of America; 6 Dipartimento di Genetica e Microbiologia, Universita' degli Studi di Bari, Bari, Italy; 7 Department of Genetics, Southwest Foundation for Biomedical Research, and Southwest National Primate Research Center, San Antonio, Texas, United States of America; Fred Hutchinson Cancer Research Center, United States of America

## Abstract

Variation in genes underlying host immunity can lead to marked differences in susceptibility to HIV infection among humans. Despite heavy reliance on non-human primates as models for HIV/AIDS, little is known about which host factors are shared and which are unique to a given primate lineage. Here, we investigate whether copy number variation (CNV) at *CCL3*-like genes (*CCL3L*), a key genetic host factor for HIV/AIDS susceptibility and cell-mediated immune response in humans, is also a determinant of time until onset of simian-AIDS in rhesus macaques. Using a retrospective study of 57 rhesus macaques experimentally infected with SIVmac, we find that *CCL3L* CNV explains approximately 18% of the variance in time to simian-AIDS (*p*<0.001) with lower *CCL3L* copy number associating with more rapid disease course. We also find that *CCL3L* copy number varies significantly (*p*<10^−6^) among rhesus subpopulations, with Indian-origin macaques having, on average, half as many *CCL3L* gene copies as Chinese-origin macaques. Lastly, we confirm that *CCL3L* shows variable copy number in humans and chimpanzees and report on *CCL3L* CNV within and among three additional primate species. On the basis of our findings we suggest that (1) the difference in population level copy number may explain previously reported observations of longer post-infection survivorship of Chinese-origin rhesus macaques, (2) stratification by *CCL3L* copy number in rhesus SIV vaccine trials will increase power and reduce noise due to non-vaccine-related differences in survival, and (3) *CCL3L* CNV is an ancestral component of the primate immune response and, therefore, copy number variation has not been driven by HIV or SIV per se.

## Author Summary

Development of vaccines for HIV/AIDS is a pressing global issue. The rhesus monkey remains the primary model for testing potential human vaccines; however, little is known about similarities and differences in host genes involved in HIV/AIDS response in humans and rhesus monkeys. Understanding these similarities and/or differences should allow more efficient testing of vaccines beneficial to humans. Here we describe the role that variation in the number of copies of *CCL3*-like genes (*CCL3L*) plays in SIV progression rates in rhesus monkeys. Copy number variation (CNV) of these genes has previously been shown to play a role in susceptibility and progression of HIV in humans. Our results suggest that individual monkeys with lower *CCL3L* copy number progress more rapidly. Accounting for *CCL3L* CNV in rhesus vaccine trials will improve researchers' abilities to interpret survival data.

## Introduction

Rhesus macaques are the most widely used non-human-primate model of HIV/AIDS [1]. We and several other research groups have reported substantial inter-individual variation in progression rates to simian-AIDS as well as population level differences between Chinese- and Indian-origin macaques [2]–[5]. Understanding the genetic basis of these individual and population differences is critical to building reliable animal models of human HIV infection and AIDS progression.

**Figure 1:**
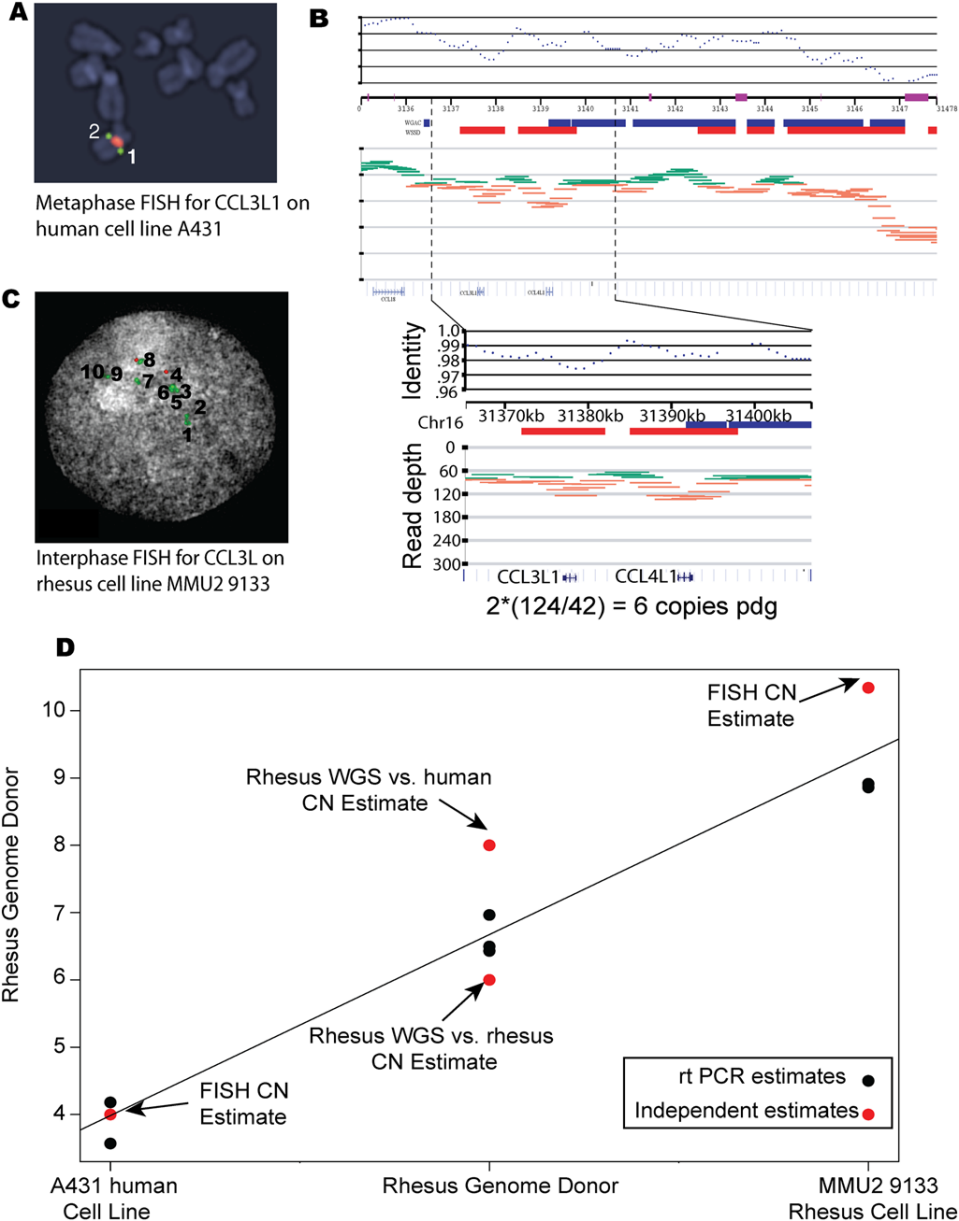
Calibration and verification of rtPCR copy number. (A) Metaphase FISH image of A431 cell line confirming diploid copy number of two *CCL3L1* genes (Note: Therefore using our assay we consider the A431 cell line to have a diploid copy number of four genes since it also contains two copies of *CCL3*). (B) Whole-genome shotgun read depth analysis showing estimation of *CCL3L* copy number in the rhesus macaque genome donor as 6 copies per diploid genome based on rheMac2 assembly. Green and orange lines denote shotgun reads aligned to *CCL3L* region of the January 2006 assembly of the rhesus macaque reference genome with orange lines showing those that likely represent regions of duplications based on the read-depth analysis (See Methods). (C) Interphase FISH image of the MMU2 9133 rhesus macaque cell line, which has an estimated diploid copy number of 10 copies of *CCL3L*. (D) Validation of rtPCR estimates of *CCL3L* copy number. Black dots represent rtPCR copy number estimates for the A431 human cell line, the rhesus genome donor, and MMU2 9133 rhesus cell line. Red dots represent an independent estimate of copy number for all three samples based on either FISH or WGS analysis. See supplemental material for additional information regarding the copy number estimation methods.

In humans, an important host factor for HIV susceptibility is copy number variation at *CCL3L1*, a paralog of the *CCL3* gene [6]–[12]. *CCL3L1* is thought to have been generated through a segmental duplication of a genomically unstable region located on human chromosome 17q11-17q12 [6]–[12]. We and others have shown that, in humans, the q arm of chromosome 17 has multiple regions of genomic instability where gene duplications, chromosomal rearrangements and copy number variation are common [13],[14]. As well, this region shows additional areas of duplication in the rhesus macaque reference genome (see Figure 1B). *CCL3* and *CCL3L1* encode chemokine ligands of *CCR5*, the main co-receptor used by HIV-1 for entry into host cells [10],[11].

After the discovery of copy number variation of *CCL3*-like genes, there have been a large number of studies in humans, expanding our understanding of the role of this variation in differential HIV susceptibility and progression. It has been shown that *CCL3*-like gene CNV plays a role in the level of chemokine production and chemotaxis [9],[15], controlling viral load [15],[16], cell-mediated immune response [17], and most recently, HIV-specific gag response [18]. Several studies have reported findings showing a significant role of *CCL3*-like gene CNV in HIV resistance and disease progression. In particular, findings indicate that reduced *CCL3L1* copy number relative to the population median correlates with increased risk of acquiring HIV [15], increased progression rate to AIDS [15], and increased risk of maternal-fetal HIV transmission [15],[19],[20]. Other studies, however, have found no, or limited, association between *CCL3*-like gene CNV and HIV/AIDS [21]–[23]. In addition, it is currently not known whether copy number variation of *CCL3-*like genes plays a role in S/HIV immunity in other primates, although it has been shown that copy number variation exists at these loci in chimpanzees [15] and that this locus is duplicated in a rhesus macaque [24].

## Results

To investigate whether *CCL3*-like genes show variable copy number in rhesus macaque populations and, more specifically, to study the role of the *CCL3*-like genes in SIV survivorship among rhesus macaques, we assayed copy number variation at these genes in a cohort of 37 Indian-origin and 20 Chinese-origin animals previously infected with SIVmac at the Tulane National Primate Research Center. Individual animals were included in our retrospective study only if the clinical results of a necropsy confirmed health complications due to simian-AIDS at the time of euthanasia or if the animal remained AIDS free for at least 18 months post-infection (see Methods and Table S1).

An analysis of the shotgun and BAC reads of the *CCL3* and *CCL3*-like gene regions of the macaque genome revealed no fixed differences that would enable us to design a *CCL3*-like gene-specific primer or probe in this species (results not shown but see Methods). Therefore, our assay, as designed, will detect both *CCL3* and all *CCL3*-like gene paralogs in rhesus as well as in chimpanzee and human cells, and we refer to the combined loci detected as *CCL3L*.

In order to estimate *CCL3L* copy numbers we used real-time PCR (rtPCR), and determined absolute copy numbers using two reference samples (see Text S1 and Figure S1 for calibration curve, and Methods for more details). The first is the human cell line A431, which has two copies per diploid genome of *CCL3* and two copies of *CCL3L1* (by fluorescent in-situ hybridization (FISH); Figure 1A; Figure S1; see also reference [9]). The second reference sample is the rhesus macaque genome donor, which whole-genome shotgun sequencing analysis (WGSA) found to have between six and eight copies per diploid genome of *CCL3L* (Figure 1B and Methods). The use of two independent references allowed us to cross-validate our copy number estimates. Support for our CNV estimates also comes from a comparison of the rtPCR result to interphase FISH of a macaque cell line (MMU2 9133) (see Figure 1C and 1D).

**Figure 2:**
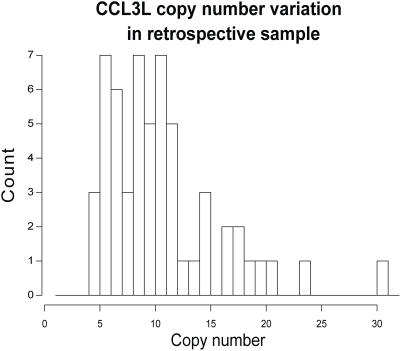
Histogram of copy number estimates. Histogram of the rtPCR estimated copy number of *CCL3*-like genes for the 57 retrospective samples of rhesus macaque. The histogram shows a large range of copy numbers found in this sample with copy number estimates from 5 to 31 copies per diploid genome.

**Figure 3:**
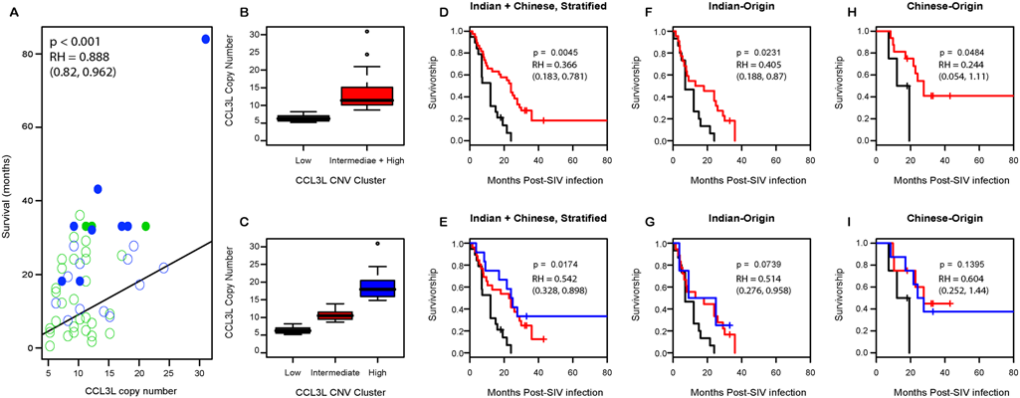
Rhesus macaque survival analysis. (A) Scatter plot of post SIV infection survival time (or censor time if animal is alive) by *CCL3L* copy number. Blue dots represent Chinese-origin rhesus macaques while green dots represent Indian origin. Filled in dots represent animals still alive at time of sampling. Fitted regression curve, *p*-value and relative-hazard (RH) from Cox proportional hazard model (model 1 in text). (B,C) Boxplots of *CCL3L* copy-number defining “low” copy number to be fewer than or equal to 8 copies per diploid genome, “intermediate” to be 9 and 14, and “high” to be more than 14 copies or low vs. intermediate+high. D-I) Estimated Kaplan-Meier survival curve for SIV-infected macaques with time measured from date of infection. The black curve represents “low”, the red curve “intermediate” or “intermediate+high”, and the blue curve “high” copy number for KM curves based on all animals (D,E), Indian-origin only (F,G), and Chinese-origin only (H,I). The *p*-values correspond to Harrington-Fleming tests of equality for survivorship curve using *ρ* = 0 which is equivalent to a log-rank or Mantel-Haenszel test. Relative-hazard (RH) for equivalent Cox proportional hazard model are also presented.

Using the rtPCR assay, we observed extensive variation in copy number of the *CCL3L* region among animals in our study, with a range of 5 to 31 copies per diploid genome (median 10; mean 11.05±5.16 [sd]; Figure 2). Tables 1 and 2 summarize the results of Cox proportional hazard models [25] for the survivorship data using *CCL3L* copy number and population-of-origin as potential covariates (see Methods). Overall, we found strong evidence that reduced *CCL3L* copy number correlates with increased rate of progression to simian AIDS. Specifically, a model that includes *CCL3L* as a covariate (*m*_1_) provides a significantly better fit to the data than the model (*m*_0_) without *CCL3L* (LRT*_m_*_0 v. *m*1_ = 11.6; *p*<0.001; Table 1; Figure 3A).

**Table 1:** Likelihood ratio test statistics for analysis of multiple variables contributing to survivorship based on Cox proportional hazard model.

Population	Model	Log-likelihood	R^2^	Model Comparison	LRT statistic	*p*-value
Combined (*n* = 57)	*m*_0_: No covariates	−155.9131	--	--	--	--
	*m*_1_: *CCL3L* copy number	−150.1400	18.3%	M1 vs. M0 (df = 1)	11.6	**<0.0007**
	*m_2_*: Population of origin	−151.7286	13.7%	M2 vs. M0 (df = 1)	8.37	**0.0038**
	*m_3_*: *CCL3L* copy number + Population of origin	−148.5120	22.9%	M3 vs. M0 (df = 2)	14.8	**0.0006**
				M3 vs. M1 (df = 1)	3.25	0.0710
				M3 vs . M2 (df = 1)	6.43	**0.0110**
Indian-only (*n* = 37)	*m*_0_: No covariates	−96.15256	--	--	--	--
	*m*_1_: *CCL3L* copy number	−93.01357	15.6%	M1 vs. M0 (df = 1)	6.28	**0.0122**
Chinese-only (*n* = 20)	*m*_0_: No covariates	−30.55236	--	--	--	--
	*m*_1_: *CCL3L* copy number	−30.07515	4.7%	M1 vs. M0 (df = 1)	0.95	0.3290

The test statistics are asymptotically χ^2^ distributed.

**Table 2:** Regression coefficient estimates (β), standard errors on the regression coefficient estimates, confidence intervals, and significance for terms in the Cox proportional hazard models summarized in Table 1.

Variable	Data	Other factors in model	β	RH	se (β)	95% CI on Exp(β)	p-value
	Combined		−0.119	0.888	0.04	(0.82, 0.96)	**0.0038**
*CCL3L*	Indian-only		−0.149	0.861	0.07	(0.76, 0.98)	**0.0260**
	Combined	Origin	−0.097	0.907	0.04	(0.84, 0.99)	**0.0220**
	Combined		−1.10	0.333	0.32	(0.18, 0.62)	**0.0006**
log_2_(*CCL3L*)	Indian-only		−1.12	0.327	0.44	(0.14, 0.77)	**0.0110**
	Combined	Origin	−0.93	0.393	0.34	(0.20, 0.76)	**0.0055**
	Combined		−0.92	0.398	0.34	(0.21, 0.77)	**0.0064**
Origin	Indian-only	*CCL3L*	−0.61	0.54	0.35	(0.27, 1.08)	0.0830
	Combined	log_2_(*CCL3L*)	−0.58	0.56	0.35	(0.20, 0.76)	0.1000

“Variable” refers to a particular term in the regression model (i.e., CCL3L copy number, log of CCL3L copy number, or population of origin), “Data” refers to which subset of the data is considered (i.e., Combined = Indian-+Chinese-origin animals or Indian animals alone), and “Other factors in the model” refer to whether the regression coefficient is estimated alone or in the presence of other terms.

Population substructure is a potential confounding variable for our analysis as it has previously been shown that Chinese-origin animals tend to exhibit slower progression rates post-infection than Indian-origin animals [2]–[5]. In order to address this issue, we first validated population assignments of all individuals in our sample by genotyping 53 unlinked microsatellites and analyzing the data using the Bayesian clustering algorithm Structure [26] and Principle Component Analysis (see Text S1; Figures S2 and S3). Both analyses clearly suggest two (and only two) sub-populations in our data with no evidence of admixture. We also calculated Queller-Goodnight [27],[28] estimates of genetic relatedness from the microsatellite data and found only low levels of cryptic relatedness within both populations (see Text S1; Figure S4). The finding that there is some level of relatedness is expected given that the animals used in our study were sampled from US colonies, however, genomic control analysis of the microsatellite data suggests that these low levels of cryptic relatedness do not markedly affect our *p*-value estimates (see Text S1; Figure S5). Once population assignments for all individuals had been confirmed, we considered several statistical models for the progression data that included population-of-origin as a potential covariate. When considered alone, we found that population-of-origin impacts survivorship with Indian-origin, correlating with increased rate of progression to simian-AIDS as previously reported (LRT*_m_*_0 v. *m*2_ = 8.37; *p*<0.01; Table 1). However, once *CCL3L* is included in the model, population-of-origin makes only a marginally significant improvement (LRT*_m_*_1 v. *m*3_ = 3.25; *p* = 0.071; Table 1). This analysis suggests that *CCL3L* is the predominant factor impacting survivorship differences among individuals, and predicts that differences in the distribution of *CCL3L* copy number among Indian and Chinese populations may explain the population-level differences in survivorship.

We further tested the impact of population substructure by repeating our analysis using only Indian-origin rhesus macaques. (The sample size and proportion of censored data in the Chinese-origin sample rendered the power of the test too low to detect a significant result; see Text S1, Figure S6). We found that including *CCL3L* CNV in the model explains a significant proportion of the survival time variation among Indian-origin macaques alone (*R^2^ = *15.6%; *p* = 0.0122), confirming that *CCL3L* CNV is contributing to the observed effect and that the effect is not likely to be explained by systematic variation at an additional allele due to population substructure. Additionally, the estimated effect size of *CCL3L* copy-number variation (*β*) on survivorship is highly comparable across subsets of the data (see Table 2 and 95% confidence intervals for exp(*β*)). This observation suggests that *CCL3L* CNV has a similar effect across both populations, whereby each copy of *CCL3L* decreases the baseline risk by a constant factor of approximately exp(*β*) = 0.907 relative to the mean of 11 copies (e.g., having 16 copies decreases the hazard by a factor of 0.907^5^  = 0.61, and having 8 copies increases the hazard by a factor 0.907^−3^ = 1.34).

Further support for the protective effects of increased *CCL3*-like gene copy number is provided by Harrington-Fleming tests of equality for Kaplan-Meier survival curves [29]. Comparisons of the survival curves across all observed *CCL3L* copy number levels clearly reject equality, whether analyzing all individuals together (*X* ^2^ = 51.3; *p*<0.001, df = 17) or stratifying by population of origin (*X* ^2^ = 48.1; *p*<0.001, df = 17). Additionally, we considered dividing the data into qualitative copy-number categories as identified by *K*-means clustering with *K* = 3 for the observed *CCL3L* CNV distribution: “low” having less than 9 *CCL3L* copies per diploid genome (pdg), “intermediate” having 9–14 *CCL3L* copies pdg, and “high” having greater than 14 *CCL3L* copies pdg. We also considered a two-class classification that combined the “intermediate” and “high” copy number classes into a single class. Overall, we observe a highly significant difference in survivorship between *CCL3L* copy classes in the combined data stratified by origin (*p* = 0.0045 for two categories and *p* = 0.0174 for three categories; see Figure 3D and 3E). Likewise, if we consider survivorship curves within each population separately, a significant difference is observed between animals with low copy number relative to those with intermediate or high copy number (*p* = 0.0231 for Indian; *p* = 0.0484 for Chinese; see Figure 3F and 3H). The above analysis is robust to how the copy-number categories are chosen. For example, using the population specific or overall first and third quartiles gives similar results (results not shown). These results, taken together, suggest that it is low *CCL3L* copy number, in particular, that is correlated with increased rate of progression.

**Figure 4:**
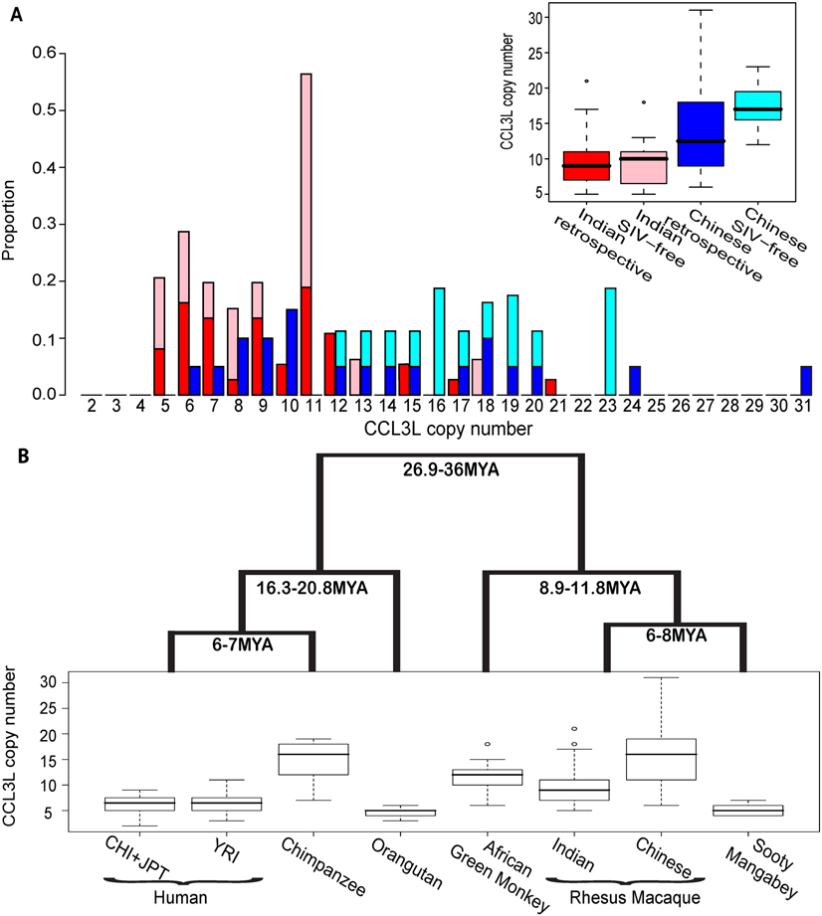
Population and species level copy number variation. (A) Histograms and boxplots of *CCL3L* copy number distribution among the *n* = 57 animals used in the retrospective study as well as for a sample SIV-free Indian-origin (*n* = 16) and Chinese-origin rhesus macaques (*n* = 15). Red and light-red bars indicate Indian origin for the SIV and SIV-free populations, and blue and light-blue bars indicate the analogous for Chinese-origin animals. B) Box plot of copy number variation for 6 primate species: Human, Chimpanzee (*Pan troglodytes*), Orangutan (*Pongo pygmaeus*), Rhesus macaque (*Macaca mulatta*), African green monkey (*Cercocebus aethiops*), and Sooty mangabey (*Chlorocebus atys*). Whiskers indicate the upper and lower quartile with dots showing outliers. Estimates of species divergence times are from reference [37].

Next we investigated whether differences in the distribution of *CCL3L* copy number alleles between populations could explain the previously reported slower simian-AIDS progression rates of Chinese-origin animals [2]–[5]. That is, given the association between higher *CCL3L* copy number and slower progression, we would expect Indian-origin macaques to have, on average, lower *CCL3L* copy numbers as compared with Chinese-origin macaques. Within the samples used for the retrospective study, animals designated as Indian-origin did, in fact, have a significantly lower mean copy number (median = 9, mean = 9.51, sd = 3.57, s.e.m = 0.587), than those designated as Chinese-origin (median = 12.5, mean 13.90, sd = 6.41, s.e.m = 1.43) as measured by a Mann-Whitney *U* test using either relative copy number estimates from rtPCR (*p* = 0.0088) or binned and rounded CNV calls (*p* = 0.0077; see also Figure 4A). We also assayed *CCL3L* CNV in an independent panel of SIV-free Indian-origin and Chinese-origin rhesus macaques to ensure that the relationship between origin and *CCL3L* was not a peculiar artifact of the animals we utilized from the SIV vaccine trials. This independent panel included 15 wild-caught Chinese-origin macaque samples collected as part of the Rhesus Macaque Genome project [24] and 16 colony-born Indian-origin macaques provided by Yerkes National Primate Center. In this second panel, we found an even higher difference in *CCL3L* CNV between the two populations (*p*<9×10^−7^ Mann-Whitney U test; also see Figure 3A). Chinese-origin animals had, on average, twice as many copies of *CCL3L* as Indian-origin animals (Chinese-origin mean = 17.6, s.d. = 3.56, s.e.m = 0.91; Indian-origin mean = 9.41, s.d = 3.4, s.e.m = 0.91), consistent with the average slower progression rates of Chinese vs. Indian-origin animals.

## Discussion

Analysis of the retrospective data provides strong support for the hypothesis that *CCL3L* CNV affects individual level SIV progression rates in rhesus macaques. This is particularly evident in the Indian-origin rhesus macaque where lower copy numbers of *CCL3L* are more common, putatively leading to an overall increase in progression rates in this population. Due to the limited power in our analysis of the Chinese-only sample, we recommend further studies to confirm the role of *CCL3L* in this population. To our knowledge, the current study provides the first example of an association between copy number variation and disease in a non-human primate. These results broaden our understanding of the role copy number variation in disease susceptibility and point to the importance of utilizing methods which allow for detecting this type of variation in genome-wide scans of disease association.

When taken together with the results of the retrospective progression study, the population level analysis suggests that differences in the distribution of *CCL3L* copy number may explain a large portion of the differences in progression rates between Indian- and Chinese-origin macaques. This result is in contrast to that found in humans, where population level differences in *CCL3L1* copy number did not translate into population level differences in progression [15]. We suggest further studies of both rhesus and human progression data is necessary to elucidate the factors contributing to these differences. Using the results of the Cox proportional hazard model, and the observed *CCL3L* distribution between subpopulations, we have generated predictions for expected survivorship at different levels of *CCL3L* copy-number variation and population-of-origin designation (provided in Text S1; Figure S7). These calculations may prove useful in the efficient design of vaccine trials. For example, we predict less than 15–20% of Indian or Chinese-origin animals with six or fewer copies of *CCL3L* will survive past 24 months post-SIV infection. In contrast, the vast majority of animals with 25 or more copies are expected to survive well past 36 months, regardless of whether they are of Indian or Chinese origin.

In this context, it is important to note that the determination of absolute copy numbers using rtPCR completely depends on the quality of the reference. Moreover, determination of absolute high copy numbers is less accurate than low copy number because noise accumulates during the progression of the amplification reaction. That said, since our absolute copy number results are based on two validated references, it is likely that they are accurate. In addition, importantly, we note that the conclusions of this study are not contingent on obtaining accurate *absolute* copy numbers for each sample. Rather, our conclusions are based on the *relative* copy number of *CCL3L* between samples, a measure that qualitatively is not sensitive to the specific reference used. Specifically, our results are robust with respect to how *CCL3L* copy number is defined. In other words, if we consider log_2_ of *CCL3L* copy number, or relative estimates of *CCL3L* copy numbers instead of absolute copy numbers, our conclusions are unchanged (Table 2).

Our findings, together with previous observations [15]–[20], suggest that *CCL3L* copy number variation is a shared genetic mechanism impacting disease progression between humans and macaques. This result is surprising given the long evolutionary time separating the two species. Population genetic theory suggests that little genetic variation currently in the human population should be shared ancestrally with rhesus macaques, so there is no *a priori* reason to suspect a shared mechanism due to a common polymorphism. As a preliminary test to determine if *CCL3L* CNV is indeed shared ancestrally, we examined *CCL3L* copy number in five other primate species: African green monkey [AGM] (*Chlorocebus atheops*, *n* = 12), sooty mangabey [SM] (*Cercocebus atys, n* = 10), orangutan [PP] (*Pongo pygmaeus*, *n* = 7), chimpanzee [PT] (*Pan troglodytes, n* = 12), and humans [HS] (8 Yoruban, 4 Chinese and 4 Japanese from the phase I HapMap set). The rtPCR was conducted using a common primer/probe set designed for the rhesus macaque. We chose to use this primer/probe set, as genomic sequence is not available for the sooty mangabey or African green monkey. Our results confirm a previous observation [15] and reveal the presence of extensive variability in *CCL3L* copy number in all primate species examined (Table 1; Figure 4B), suggesting that *CCL3L* CNV has likely been segregating in Old World monkeys and apes for at least 25 million years through recurrent duplication, deletion, and gene conversion of the locally unstable genomic segments containing the *CCL3L* genes.

We note, however, that due to the use of a common primer/probe set in all species, the determination of absolute copy number may be biased by fixed differences between species in these regions. This is in contrast to the observation that copy number is variable within each species, which should be robust to such fixed differences. We also note that our results differ from those of a recent study of genomic copy number variation in chimpanzees and humans, which found low *CCL3L* copy numbers in chimpanzees [30]. It is important to highlight that there are likely differences in the subspecies of chimpanzee used between the studies and in the resolution of the CNV detection methods utilized. These experimental design differences make it difficult to draw conclusions regarding the biological significance of the differing observations. We therefore suggest that more research is needed to resolve the evolutionary history of this genomic region, and in particular to estimate the distribution of *CCL3L* copy number variation in chimpanzees.

In summary**,** our findings further support the hypothesis that *CCL3L* copy number variation is an important host factor for explaining variation in HIV/SIV progression rates [15]–[20]. Our results also provide an example of a common mechanism of increased survival time after infection with HIV or SIV in humans and another primate species respectively. There are two immediate predictions from our observations. First, stratifying by *CCL3*-like gene copy number in macaque vaccine studies will allow researchers to remove *CCL3L* as a confounding effect, thereby increasing the power of vaccine trials. Second, based on our observations, we suggest that rhesus macaque is a valuable model organism for further studies of the specific mechanism by which *CCL3*-like gene copy number affects rates of HIV progression in humans.

Finally, it is important to note some caveats of our work. First, the current study is based on a relatively small sample of rhesus macaques, pooled across several SIV studies. Additional data is needed to fully understand the role of *CCL3L* in rhesus macaque SIV progression. For example, further analysis investigating the role of *CCL3L* on viral load and CD4-T cell count levels would be beneficial. Unfortunately, complete and consistently taken measurements are not available for these data as the samples were pooled across several experiments. Second, as with all studies using rtPCR technology, it is possible that polymorphisms in the primer and probe sites could affect copy number estimation–although our sequencing effort of the PCR products from four samples suggests that the presence of such polymorphisms is unlikely. Additionally, the presence of pseudogeninzed copies may bias our results. Unfortunately, without complete knowledge of the presence and distribution of pseudogenes for each individual, it is difficult to address how the presence of pseudogenes would impact the power of the analysis. For example, if for each monkey 1–2 copies of *CCL3*-like genes are pseudogenized, this would have little (or no) impact on the power of the tests. However, if for example, as copy number increases, the probability of having more pseudogenized copies also increases, this would adversely impact the power of the test.

Third, we have used a candidate gene approach in our analysis of the association of genetic variation with simian-AIDS progression. Therefore, it is possible that genetic variants at other loci may account for even larger portion of the variance in survival. One possible example is variation at MHC class I genes, which has been shown to be associated with SIV progression rate in Indian-origin rhesus macaques and explains at least 48% of the variance in that study [31]. We have addressed this to some extent by conducting replicate association analyses with the 53 genome-wide, unlinked microsatellite loci (see Text S1; Figure S5). We find that copy number variation at the *CCL3L* locus falls in the 1% tail of this distribution (after accounting for population substructure) and is therefore, likely a true positive. It is important to remember that there are many other host factors aside from chemokines and their receptors known to influence HIV susceptibility and pathogenesis in humans [32],[33]. We believe these other factors should be characterized in rhesus along with discovery of rhesus-specific genetic variation before conclusions can be drawn on the relative importance of shared versus species-specific factors influencing retroviral susceptibility and disease progression.

## Methods

### Retrospective Progression Samples

The rhesus macaques used in the retrospective analysis were all inoculated with SIVmac as part of previous SIV research programs at the Tulane National Primate Research Center. All macaques were infected with SIVmac239 or SIVmac251 with SIV inoculum given under a standard protocol and at similar mid-level dose. Doses in this range and the strain used have been previously shown not to affect the outcome of disease course [34].

All animals used in our study were euthanized under the same set of guidelines if they did not remain healthy after infection. Specifically, euthanasia was carried out if life threatening clinical conditions indicated that the life expectancy of the animal was less than 7 days. Following euthanasia, a necropsy was performed, and animals were only included in the current study if the necropsy confirmed SIV as the underlying cause of the clinical state. Animals were excluded only if they were euthanized and illness could not be confirmed to be AIDS related. Because the results of the necropsy were inconclusive with respect to the cause of the illness in these cases (SIV or not) we chose to exclude them. Conducting the analyses with these animals included as either censored data or non-censored data did not change the results of the analysis. Under these protocols, the time of euthanasia will give a reasonable approximation to both time to progression to simian-AIDS and survival time, as the presence of AIDS defining illnesses met the criterion for euthanasia. (See Table S1 for clinical findings of necropsy and Text S1).

### Additional Primate Samples

DNA extractions from the uninfected Chinese-origin rhesus samples were obtained from the Rhesus macaque genome consortium. The chimpanzee, orangutan, sooty mangabey, and uninfected Indian-origin rhesus macaque DNA samples were obtained from the Yerkes National Primate Research Center and the African green monkey DNA samples were obtained from the University of California Los Angeles.

### Real-Time PCR *CCL3L* Copy Number Estimation

*CCL3L* gene copy number was determined using real-time quantitative PCR (rtPCR) on a 7900HT Fast Real-Time PCR System (Applied Biosystems Inc.) with the JumpStart *Taq* ReadyMix (SIGMA) and *TaqMan* probes. The PCR included 18 ng total genomic DNA. Cycling conditions were: initial denaturation at 94°C for 2 min; followed by 40 cycles of 15 sec denaturation at 94°C and 1 minute annealing/extension at 60°C. The *Stat6* gene, found to be present in a single copy, per haploid genome, in rhesus macaque, chimpanzee and human reference genomes, was used as the internal control. Oligonucleotide sequences used for *CCL3L* were: Forward: 5′-CCAGTGCTTAACCTTCCTCC-3′, Reverse: 5′–TCAGGCACTCAGCTCCAGGT-3′, Probe: 5′-AGGCCGGCAGGTCTGTGCTGACC-3′. For *Stat6*, sequences were: Forward: 5′-CCAGATGCCTACCATGGTGC-3′, Reverse: 5′-CCATCTGCACAGACCACTCC-3′, Probe: 5′-CTGATTCCTCCATGAGCATGCAGCTT-3′. This primer set does not distinguish between *CCL3* and the *CCL3*-like gene paralogs, as we did not observe sufficient fixed differences between these paralogs in rhesus macaque references genome to design a specific assay. It is also unknown whether any pseudogenized copies of *CCL3L* genes exist in the rhesus macaque populations. As such, we here refer to *CCL3* and its paralogs as *CCL3L*. PCR results were analyzed using SDS v2.2.1 software package (Applied Biosystems Inc.). We performed rtPCR for each individual in triplicate and determined the normalized *relative* copy number by generating a standard curve and then normalizing across samples by the results of the *Stat6* control gene and dividing the value obtained by one of the reference individuals.

### Analysis of *CCL3L* Copy Number Based on Reference Samples

To estimate the absolute *CCL3L* copy number for each sample based on the rtPCR results described above, we used two reference samples: the A431 human cell line and the rhesus genome donor individual. The A431 cell line was chosen as it has previously been shown to have two copies of *CCL3L1* and two copies of *CCL3* pdg [9], for a total copy number of four *CCL3L* using the rtPCR assay described above. To confirm the *CCL3L1* copy number of the particular A431 cell line culture used here, we performed florescent in situ hybridization (FISH) of metaphase chromosomes using the human fosmid probes WIBR2-3688L07 (*CCL3L1* specific; green spots on Figure 1A) and WI2-653M1 (chr. 17 single copy control; red spots on Figure 1A). Visualization of the FISH assay clearly shows that this cell line extract had 2 copies of *CCL3L1* pdg.

The second reference sample was the rhesus macaque genome donor sample. Copy number of the *CCL3L* locus for this sample was determined using whole genome shotgun (WGS) read depth analysis [24],[35]. Read depth analysis was performed by aligning all fragments of minimum 150 bp of non-repeat masked sequence to the to the macaque *CCL3L1* locus with a 95% identity threshold. We compared the average depth of WGS sequence coverage for unique (not-duplicated) sequence in 5kb windows with the depth of coverage to the *CCL3L1* locus to estimate copy-number of the locus (Figure 1B). The experiment was repeated using the human *CCL3L* locus as a reference with an 88% identity threshold (results not shown). From these analyses, we predicted the *CCL3L* copy number for the genome donor macaque to be 6–8 copies of *CCL3L* pdg depending on whether the rhesus or human genome is used for alignment. The difference in estimated copy number between the alignment to the rhesus genome and that of the human genome is likely due to alignment of non-*CCL3L* genes. Due to this, alignment to the rhesus genome is likely a better predictor of *CCL3L* copy number for this individual because it is less likely to include non-*CCL3*-like gene paralogs.

We determined the absolute *CCL3L* copy number in each sample by comparing rtPCR results between samples and the references. Specifically, the normalized rtPCR values were averaged across the three replicates for each individual and divided by the averaged rtPCR results for one of the reference samples and multiplied by 4 (the diploid copy number of the A431 cell line including *CCL3L*1 and *CCL3*) or 7 (the average diploid copy number of the rhesus macaque donor individual). The resulting number was then rounded to the nearest integer value to estimate absolute copy number. In Figure S1, we report the calibration curves for the A431 reference samples and demarcation of inferred copy number pdg for each sample. All statistical analyses were conducted using the rounded as well as the raw values.

### Confirmation of rtPCR *CCL3L* Copy Number Estimate

To confirm that the rtPCR absolute copy number estimates were accurate we estimated *CCL3L* copy number for an additional rhesus macaque cell line using both rtPCR and interphase FISH (Figure 1C). The rtPCR estimated diploid copy number for this macaque cell line is 9 using either reference sample. The estimated *CCL3L* copy number from the FISH experiment is 10.34±3.00 (mean±standard error based on 54 replicate FISH experiments). The slight discrepancy between the rtPCR and FISH is likely due to the fact that the FISH probe used contains other, known, structural variants which show higher copy number in the macaque reference genome (visible in WGS read depth analysis see Figure 1C). As well, the proximity of the *CCL3L* gene copies renders it difficult to distinguish distinct copies in some of the FISH images.

### Primers in Additional Species

The same rtPCR primers and probe were used in all primate species. These primers are not specific to the other species and differences in both the chimpanzee and human reference priming sequences were observed. We note that at the time of this study no reference sequences were available for the sooty mangabey or the African green monkey on which to design species-specific probes. While this may lead to slight biases in the determination of the absolute copy number for any particular individual or species, it does not effect the overall conclusions of the study that all species surveyed show population level variation in copy numbers.

### Statistical Analysis

All statistical analysis was conducted using the R statistics package. Significance of copy number differences between Indian-origin and Chinese-origin populations of SIV and non-SIV infected rhesus macaque was evaluated using a Mann-Whitney U test. Survival analyses of the SIV infected macaque data were conducted using the survival package in R.

The Cox proportional hazard model was chosen, as it is a flexible semi-parametric regression model that accounts censored data. Let *i* = 1…*n* index individuals and *j* = 1…*p* index variables of the regression model. The Cox proportional hazard rate of individual *i* at time *t* has the form:
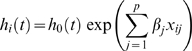
where *h_0_*(*t*) is the base line hazard function, the *x_ij_*'s for *j* = 1…*p* are the covariates for individual *i*, and the β*_j_*'s are regression coefficients. An underlying assumption of this model is that the covariates act additively on the log of the hazard function and that the log hazard function changes linearly with the β terms. These are referred to as the proportionality assumptions. We tested this assumption using the method proposed by Grambsch and Therneau [36] as implemented in the survival package in R and found that the assumption holds for these data. It is important to note that there no assumption is made regarding the functional form of base line hazard function *h_0_*(*t*). The reason for this is that our object of analysis is the *proportional hazards* among individuals that at time *t* are independent of *h_0_*. For example, considering a pair of individuals *i* and *i*′, the hazard ratios are:
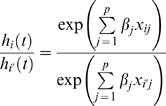



The model parameters β_1_…β_p_ are estimated given the *ranked* observed failure times *y_1_*<*y_2_*<…<*y_n_* using the partial likelihood method proposed by Cox [25] as implemented in the coxph function in R. Since some data are censored, we introduce *n′* to denote the number of uncensored observations. The partial likelihood is given by:
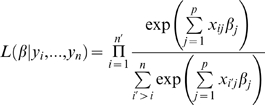



Four models are considered; *m*_0_, which includes no covariates; *m*_1_, which includes only *CCL3L* copy number as a potential covariate; *m*_2_, which considers only population-of-origin as a factor, and *m*_3_, which considers both *CCL3L* copy number and population of origin. To choose among nested regression models for the SIV infected macaque survival data, we used twice the difference in log-likelihood and assessed significance using standard χ^2^ approximations.

The Harrington and Fleming procedure was used to assess differences among Kaplan-Meier survival curve. This method was also implemented in the survdiff function of the R survival package. All analysis labeled “stratified” were conducted by including the term strata(origin) in the right hand side of the regression equation where origin is an indicator variable of Chinese-origin (i.e., 1 if Chinese, 0 if Indian). The survfit routine to generate predicted Kaplan-Meir survival curves as a function of *CCL3L* copy number and population-of-origin. All R scripts used for analysis and production of figures are available from the investigators upon request.

## Supporting Information

Figure S1 Calibration curve for rtPCR assay using A431 cell line as a standard. Since the A431 cell line has four copies of *CCL3L* (see Figure 1A), *CCL3L* copy number is inferred as the relative rtPCR level for a sample, multiplied by 4 and rounded to the nearest integer. Each color represents a transition in copy number variation call (i.e., the break between 5 copies and 6 copies is denoted by a transition of red to green, and the break between 6 and 7 copies by a transition from green to dark blue).
(0.81 MB TIF)

Figure S2 Structure results of the retrospective individuals from the 53 microsatellite loci sorted by assumed population. Red are Indian origin animals and blue are Chinese origin animals.
(1.07 MB TIF)

Figure S3 PCA results for the retrospective sample. Red are Indian origin and blue are Chinese origin. (A) Box-plot of PC1 values. (B) Bi-plot of PC1 vs. PC2 showing distinct clustering of animals into proper sub-populations.
(1.19 MB TIF)

Figure S4 Heat plots summarizing genetic relatedness in the sample based on 53 unlinked microsattelite loci. (A) Pearson product-moment correlation of genotypic state for all individuals in the sample; (B) Queller-Goodnight r distance between pairs of individuals in the Indian-origin sample; (C) QG distances for individuals in the Chinese-origin sample.
(4.89 MB TIF)

Figure S5 (A) Quantile-Quantile plot of the empirical *p*-value distribution from the 53 unlinked microsatellites versus that expected under a uniform distribution. (B) Histogram of the −log10 *p*-values from the microsatellite data with arrow showing the position of the *p*-value for the association with log2 *CCL3L* copy number and survival.
(1.00 MB TIF)

Figure S6 Bootstrap simulations to assess power of Cox proportional hazard regression of survivorship on *CCL3L* copy number applied to each population separately.
(0.57 MB TIF)

Figure S7 Predicted Kaplan-Meier survival curves based on Cox Proportional hazard model of post-SIV survivorship including *CCL3L* copy number and population-of-origin as covariates. Dashed lines indicate 95% prediction intervals based on application of the function survfit in the survival R package.
(0.88 MB TIF)

Table S1 Results of necropsy results for 57 animals used in the retrospective study.
(0.06 MB PDF)

Table S2 Total number of polymorphic sites found per primer/probe/individual for *CCL3L* rtPCR assay. CH1 and CH2 are two macaque individuals of Chinese origin. IN1 and IN2 are Indian-origin macaques.
(0.04 MB PDF)

Table S3 Summary statistics for *CCL3L* copy number distribution among primate species and populations.
(0.05 MB PDF)

Table S4 Microsatellite id, number of alleles found in the retrospective sample, and heterozygosity for the 53 typed microsatellites.
(0.04 MB PDF)

Text S1 Additional methods describing validation of rtPCR primers and probes, analysis of microsatellite data, and power analysis.
(0.05 MB DOC)
