# Supplementary figures and images for: Copy Number Variation of CCL3-like Genes Affects Rate of Progression to Simian-AIDS in Rhesus Macaques (Macaca mulatta)
Source: PLoS Genet. 2009 Jan 23;5(1):e1000346. doi: 10.1371/journal.pgen.1000346 (PMC2621346; doi:10.1371/journal.pgen.1000346)

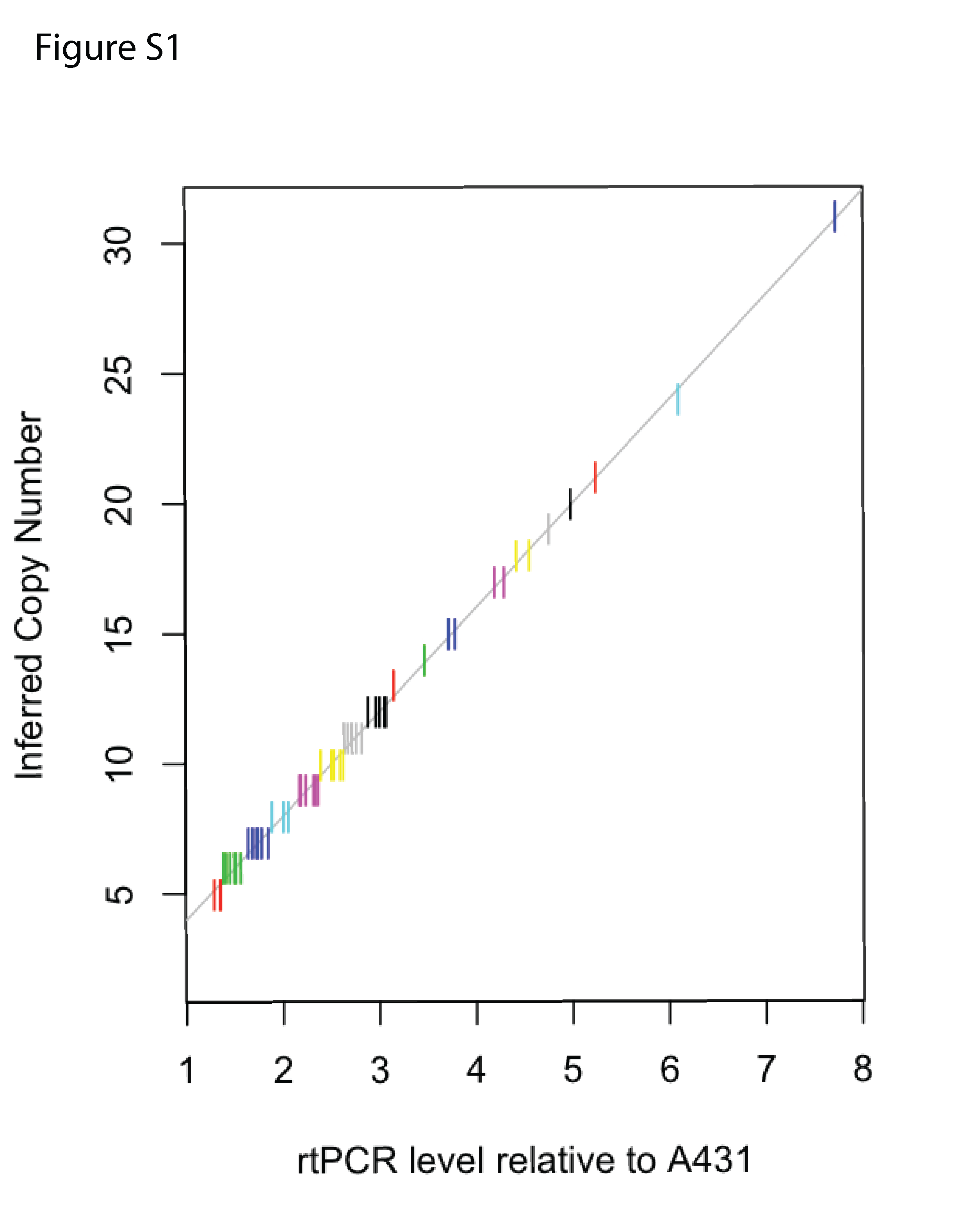

Supplement: Figure S1 — Calibration curve for rtPCR assay using A431 cell line as a standard. Since the A431 cell line has four copies of CCL3L (see Figure 1A), CCL3L copy number is inferred as the relative rtPCR level for a sample, multiplied by 4 and rounded to the nearest integer. Each color represents a transition in copy number variation call (i.e., the break between 5 copies and 6 copies is denoted by a transition of red to green, and the break between 6 and 7 copies by a transition from green to dark blue). (0.81 MB TIF) [file pgen.1000346.s001.tif]

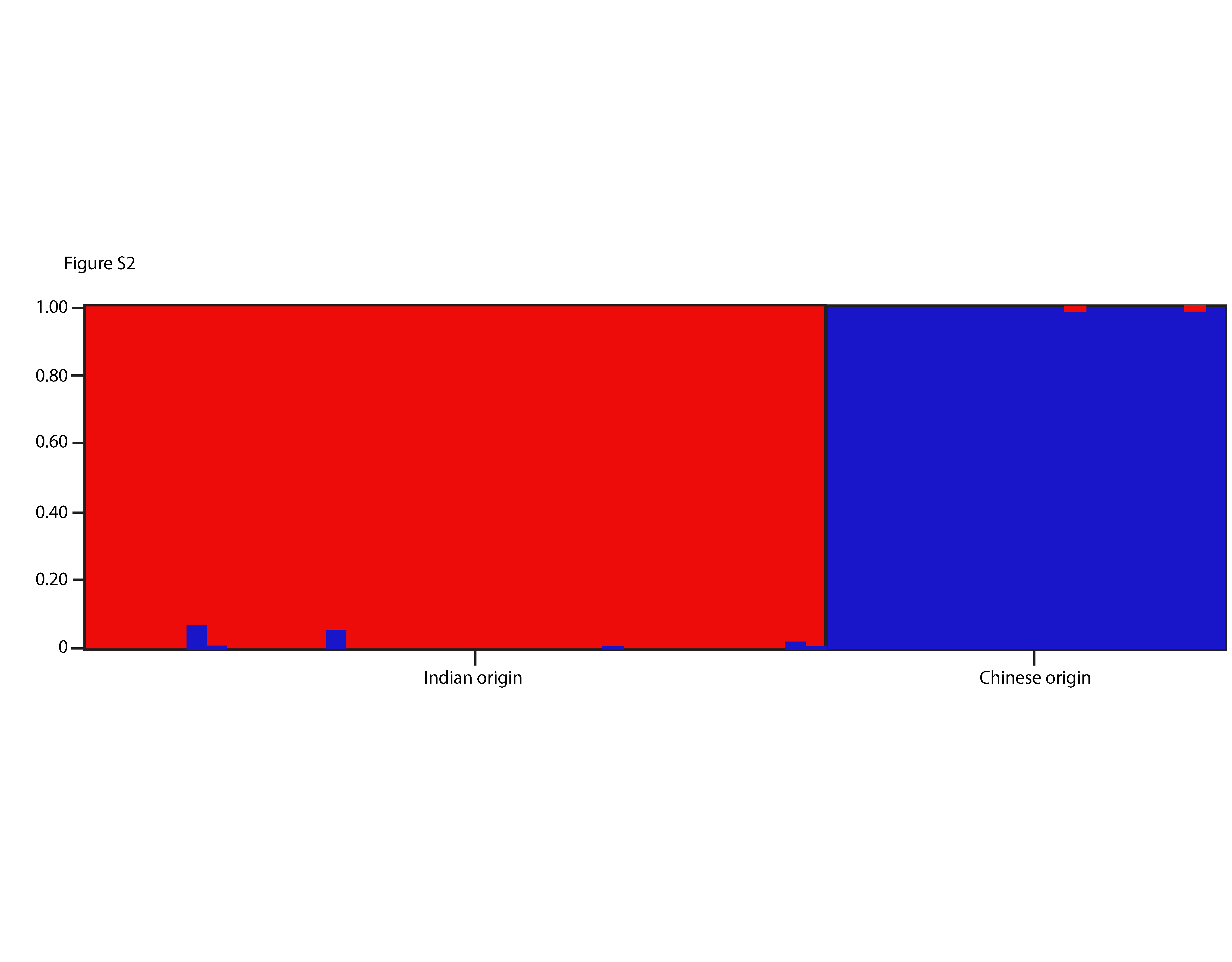

Supplement: Figure S2 — Structure results of the retrospective individuals from the 53 microsatellite loci sorted by assumed population. Red are Indian origin animals and blue are Chinese origin animals. (1.07 MB TIF) [file pgen.1000346.s002.tif]

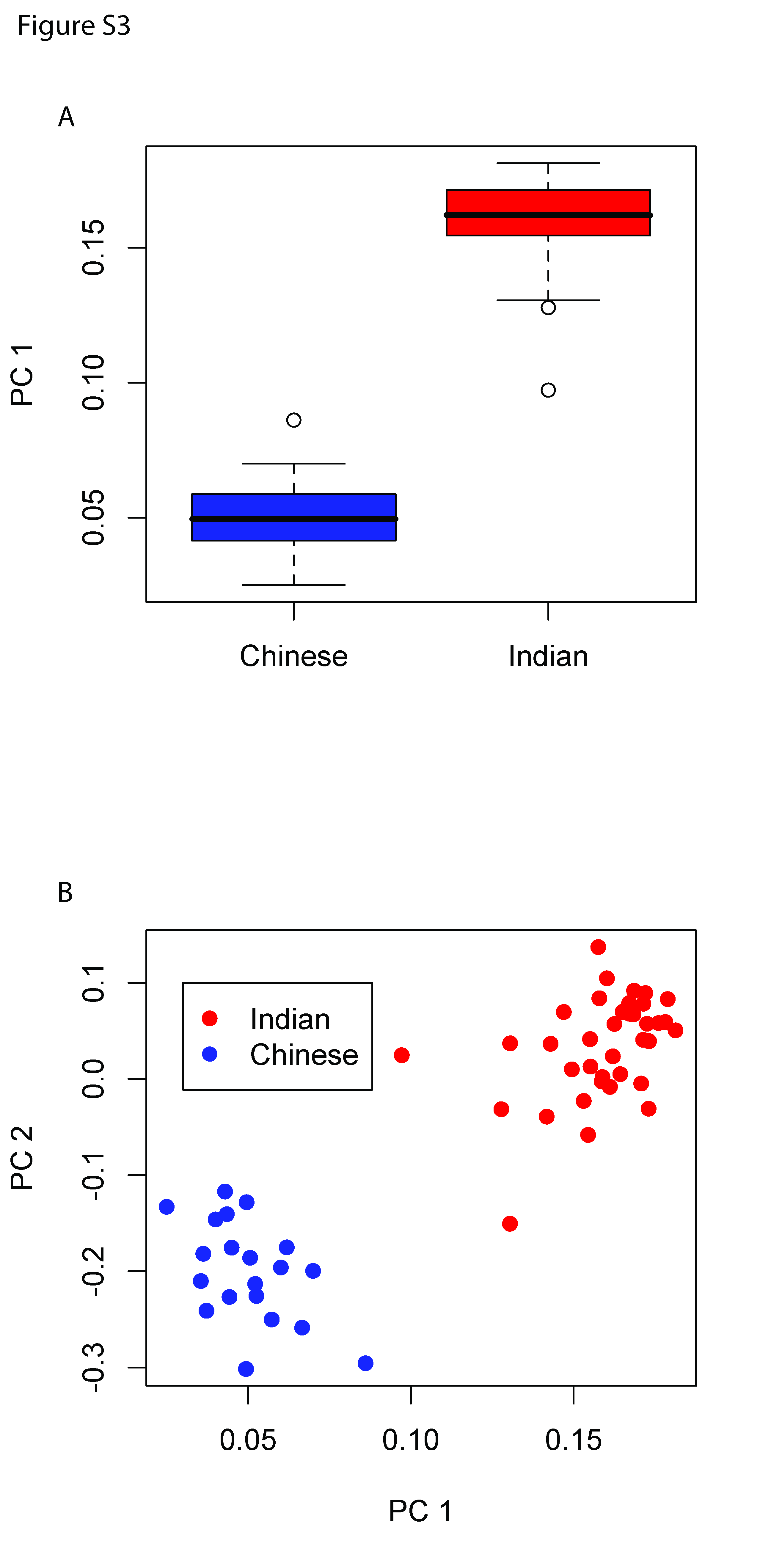

Supplement: Figure S3 — PCA results for the retrospective sample. Red are Indian origin and blue are Chinese origin. (A) Box-plot of PC1 values. (B) Bi-plot of PC1 vs. PC2 showing distinct clustering of animals into proper sub-populations. (1.19 MB TIF) [file pgen.1000346.s003.tif]

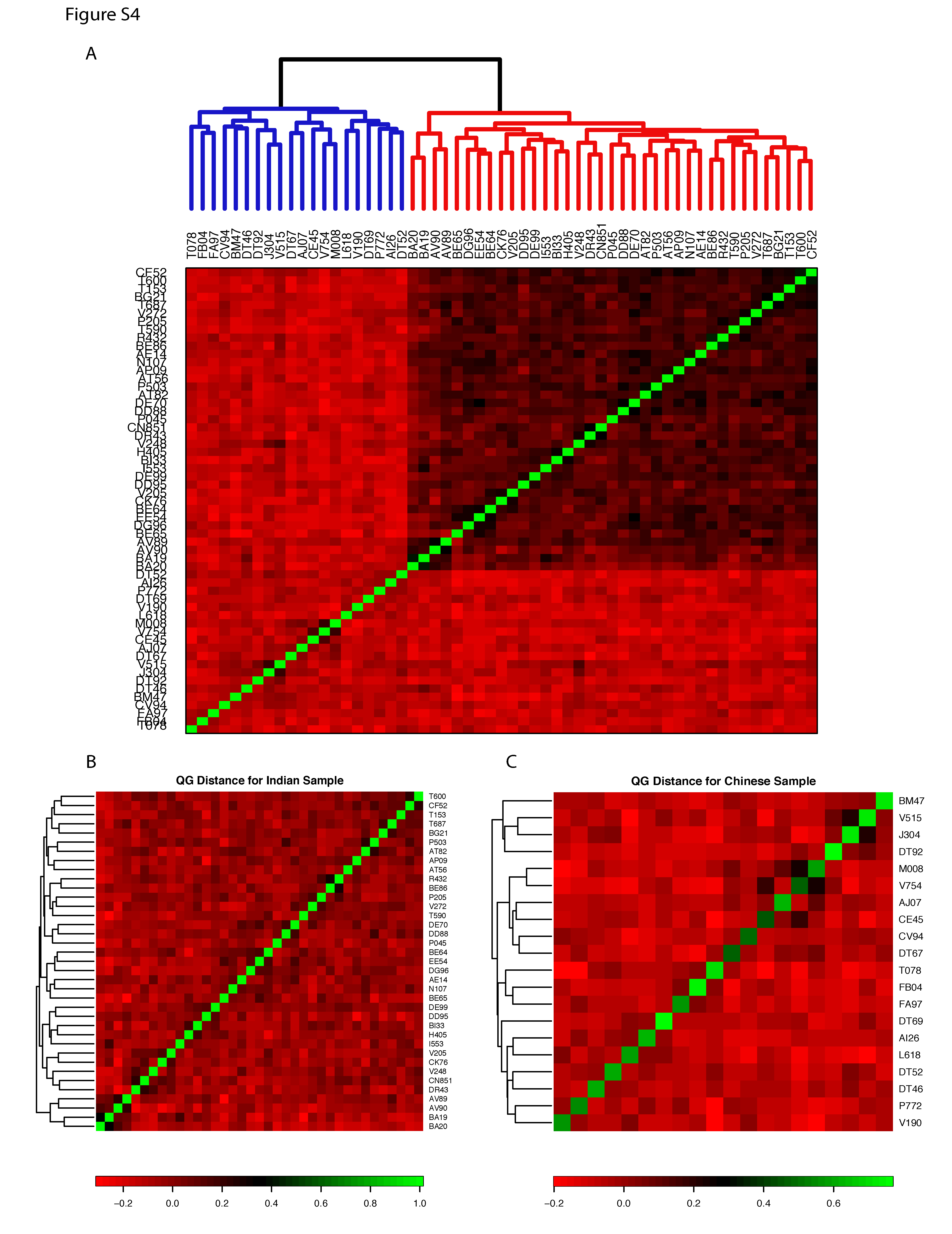

Supplement: Figure S4 — Heat plots summarizing genetic relatedness in the sample based on 53 unlinked microsattelite loci. (A) Pearson product-moment correlation of genotypic state for all individuals in the sample; (B) Queller-Goodnight r distance between pairs of individuals in the Indian-origin sample; (C) QG distances for individuals in the Chinese-origin sample. (4.89 MB TIF) [file pgen.1000346.s004.tif]

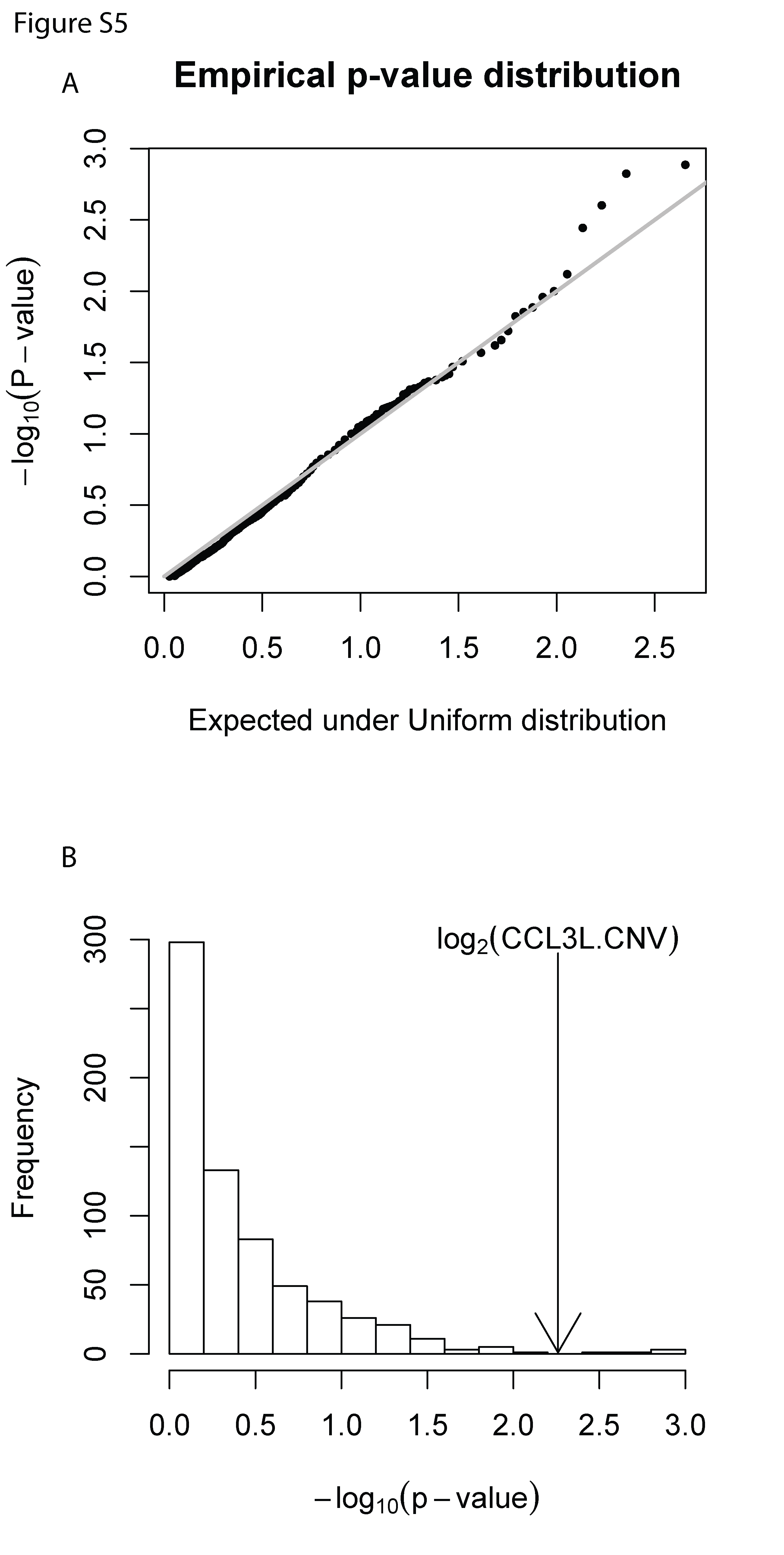

Supplement: Figure S5 — (A) Quantile-Quantile plot of the empirical p-value distribution from the 53 unlinked microsatellites versus that expected under a uniform distribution. (B) Histogram of the −log10 p-values from the microsatellite data with arrow showing the position of the p-value for the association with log2 CCL3L copy number and survival. (1.00 MB TIF) [file pgen.1000346.s005.tif]

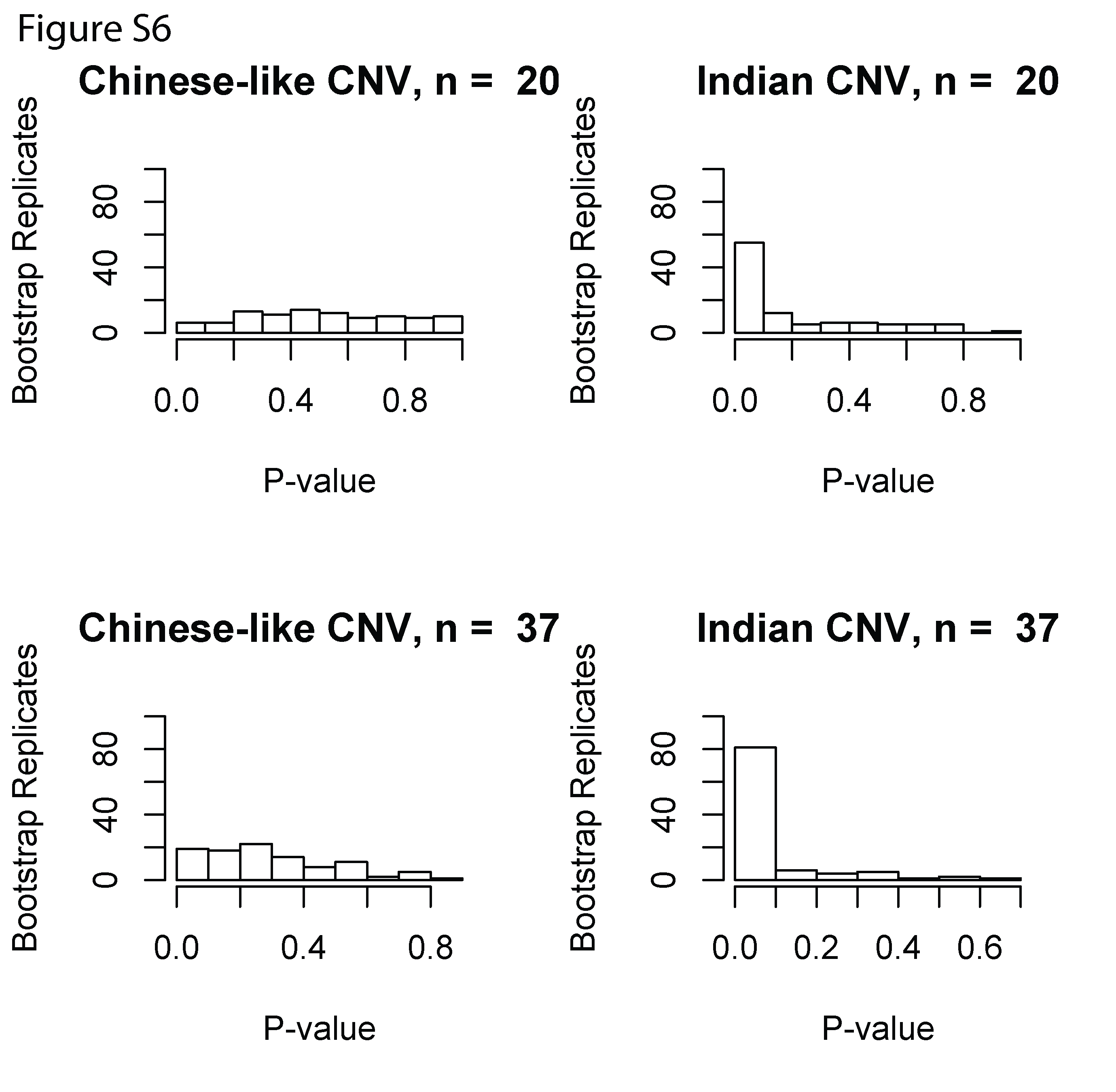

Supplement: Figure S6 — Bootstrap simulations to assess power of Cox proportional hazard regression of survivorship on CCL3L copy number applied to each population separately. (0.57 MB TIF) [file pgen.1000346.s006.tif]

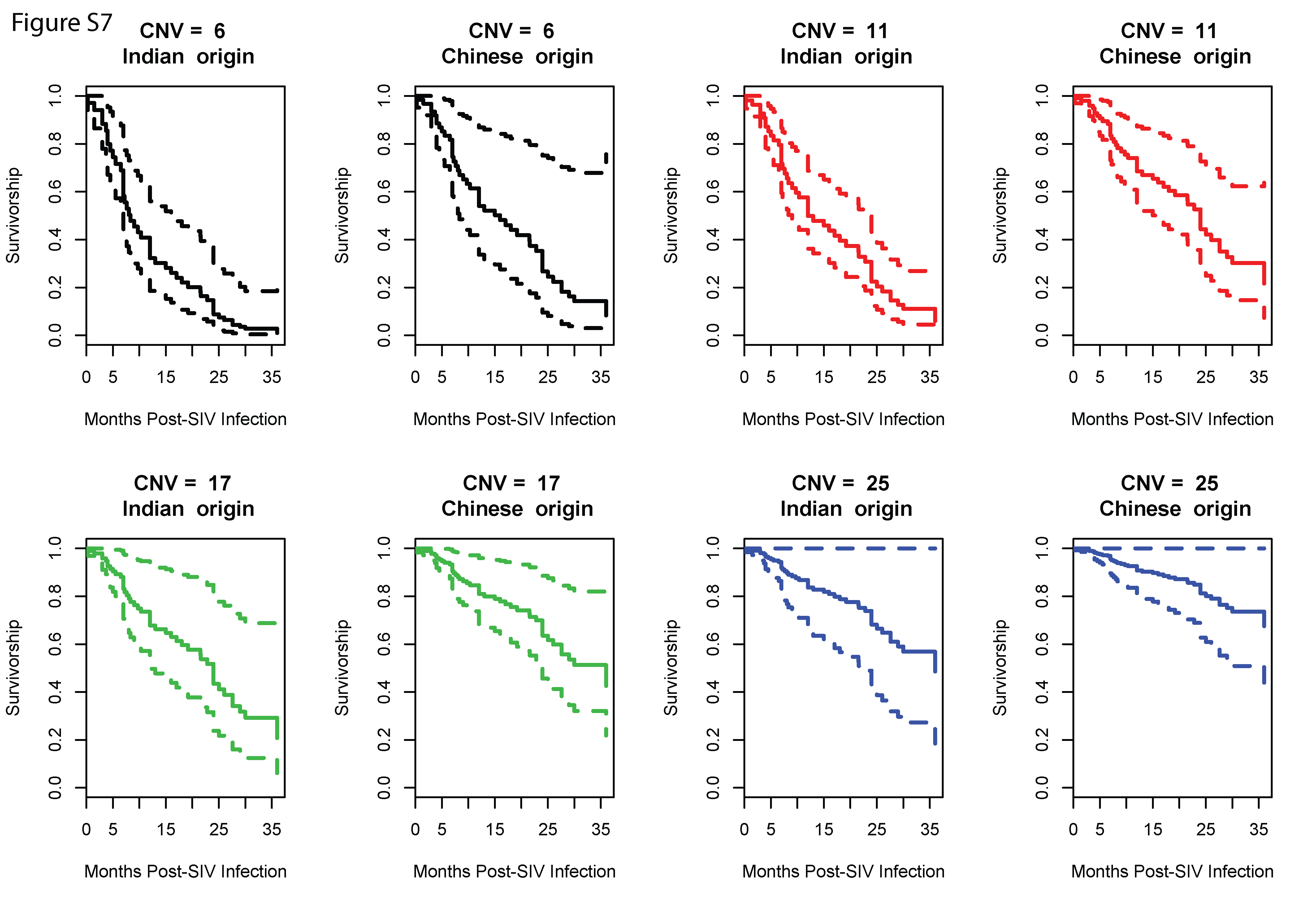

Supplement: Figure S7 — Predicted Kaplan-Meier survival curves based on Cox Proportional hazard model of post-SIV survivorship including CCL3L copy number and population-of-origin as covariates. Dashed lines indicate 95% prediction intervals based on application of the function survfit in the survival R package. (0.88 MB TIF) [file pgen.1000346.s007.tif]
