# Supplementary material for: Copy Number Variation of CCL3-like Genes Affects Rate of Progression to Simian-AIDS in Rhesus Macaques (Macaca mulatta)
Source: PLoS Genet. 2009 Jan 23;5(1):e1000346. doi: 10.1371/journal.pgen.1000346 (PMC2621346; doi:10.1371/journal.pgen.1000346)
Supplement: Table S1 — Results of necropsy results for 57 animals used in the retrospective study. (0.06 MB PDF) [file pgen.1000346.s008.pdf]

**Table S1**: Results of necropsy results for 57 animals used in the retrospective study.

| Animal | Survival time (months) | Clinical findings of necropsy (if not alive) | Origin |
|---|---|---|---|
| V272 | .27 | Severe lymphoid hyperplasia of small intestine lymphoid follicle | Indian |
| BA20 | 1.5 | Hepatic steatosis, gastroenterocolitis | Indian |
| AT56 | 3 | Amyloidosis, enterocolitis | Indian |
| BA19 | 3 | Colitis (SIV) | Indian |
| T600 | 3.7 | Cytomegalovirus infection | Indian |
| DE99 | 4 | SIV infection | Indian |
| T590 | 4 | Opportunistic infection | Indian |
| H405 | 4.5 | Colitis, pancreatic amyloidosis | Indian |
| DG96 | 5 | Pneumonia, CMV, cryptosporidium | Indian |
| R432 | 5.5 | Giant Cell Disease (SIV) | Indian |
| AV90 | 6.5 | Colitis, pneumonia, encephalitis, giant cell | Indian |
| AJ82 | 7 | Enterocolitis | Indian |
| BE86 | 7 | SIV infection | Indian |
| DE70 | 7 | Pneumocystis, colitis, giant cell | Indian |
| DR43 | 7 | SIV infection | Indian |
| DT52 | 7.2 | Colitis (SIV) | Chinese |
| P205 | 7.6 | Amyloidosis/intestinal | Indian |
| AE14 | 8 | Enteritis (SIV) | Indian |
| DT69 | 8.3 | Pneumonia | Chinese |
| N107 | 9 | Cryptosporidium infection | Indian |
| DT67 | 9.7 | Pneumonia | Chinese |
| FA97 | 10.3 | Cytomegalovirus infection | Chinese |
| CK76 | 12 | Lymphoid hyperplasia, Giant cell | Indian |
| CV94 | 12 | Pneumocystis carinii | Chinese |
| DD88 | 12 | Amyloidosis, colitis | Indian |
| EE54 | 12 | SIV infection, pneumocystis | Indian |
| CN851 | 13 | Pulmonary infarct, pneumonia, colitis | Indian |
| DD95 | 15 | SIV infection | Indian |
| CF52 | 16 | Lymphoid hyperplasia | Indian |
| CE45 | 17 | Pneumonia | Chinese |
| FB04 | 18 | Alive at time of Sampling | Chinese |
| V248 | 18 | Colitis, amyloidosis (SIV) | Indian |
| V754 | 18 | Alive at time of Sampling | Chinese |
| J304 | 19.2 | Neoplasm/Lymphoma | Chinese |
| I553 | 21.5 | Pneumonia | Indian |
| V515 | 21.6 | Mycobacterium avium/intracellular | Chinese |
| AL26 | 22.8 | Pneumonia/interstitial | Chinese |

| | | | |
|---|---|---|---|
| P503 | 24 | Lymphoma, hepatic amyloidosis, opportunistic infection | Indian |
| T687 | 24 | Undetermined but SIV related | Indian |
| V190 | 24 | Mycobacterium avium/intracellular | Chinese |
| V205 | 24 | Intestinal amyloidosis | Indian |
| BE64 | 25 | Pericarditis, vasculitis, cardiac and pulmonary thrombi | Indian |
| P045 | 26 | Pneumocystis pneumonia, giant cell | Indian |
| P772 | 27.6 | Pneumocystis carni infection | Chinese |
| T078 | 27.6 | Mycobacterium avium/intracellular | Chinese |
| AV89 | 29 | Lymphoma | Indian |
| BI33 | 30 | Pneumocystis pneumonia | Indian |
| M008 | 32 | Alive at time of Sampling | Chinese |
| AP09 | 33 | Alive at time of Sampling | Indian |
| BG21 | 33 | Alive at time of Sampling | Indian |
| BM47 | 33 | Alive at time of Sampling | Chinese |
| DT46 | 33 | Alive at time of Sampling | Chinese |
| L618 | 33 | Alive at time of Sampling | Chinese |
| T153 | 33 | Alive at time of Sampling | Indian |
| BE65 | 36 | Mycobacterium avium infection | Indian |
| DT92 | 43 | Alive at time of Sampling | Chinese |
| AJ07 | 84 | Alive at time of Sampling | Chinese |
