# Supplementary material for: Copy Number Variation of CCL3-like Genes Affects Rate of Progression to Simian-AIDS in Rhesus Macaques (Macaca mulatta)
Source: PLoS Genet. 2009 Jan 23;5(1):e1000346. doi: 10.1371/journal.pgen.1000346 (PMC2621346; doi:10.1371/journal.pgen.1000346)
Supplement: Table S2 — Total number of polymorphic sites found per primer/probe/individual for CCL3L rtPCR assay. CH1 and CH2 are two macaque individuals of Chinese origin. IN1 and IN2 are Indian-origin macaques. (0.04 MB PDF) [file pgen.1000346.s009.pdf]

Table S2. Total number of polymorphic sites found per primer/probe/individual for *CCL3L* rtPCR assay. CH1 and CH2 are two macaque individuals of Chinese origin. IN1 and IN2 are Indian-origin macaques.

Individual	Forward primer	Reverse Primer	Probe
CH1	1	4	4
CH2	1	2	0
IN1	3	1	1
IN2	0	0	0
