# Supplementary material for: Copy Number Variation of CCL3-like Genes Affects Rate of Progression to Simian-AIDS in Rhesus Macaques (Macaca mulatta)
Source: PLoS Genet. 2009 Jan 23;5(1):e1000346. doi: 10.1371/journal.pgen.1000346 (PMC2621346; doi:10.1371/journal.pgen.1000346)
Supplement: Table S3 — Summary statistics for CCL3L copy number distribution among primate species and populations. (0.05 MB PDF) [file pgen.1000346.s010.pdf]

Table S3. Summary statistics for *CCL3L* copy number distribution among primate species and populations.

Population	*n*	Mean	SD	Median
Human (Yoruban [YRI])	8	6.125	2.17	6.5
Human (Chinese [CHI]+ Japanase [JPT])	8	6.5	2.45	6.5
Chimpanzee	12	14.58	4.27	16
Orangutan	7	4.57	0.976	5
Rhesus macaque (Chinese – SIV free)	15	17.6	3.56	17
Rhesus macaque (Chinese – SIV retrospective)	20	13.9	6.40	12.5
Rhesus macaque (Indian – SIV free)	16	9.44	3.4	10
Rhesus macaque (Indian – SIV retrospective)	37	9.41	3.56	9.0
African green monkey	12	11.58	3.15	12
Sooty Mangabey	10	5.2	1.32	5
