# Supplementary material for: Copy Number Variation of CCL3-like Genes Affects Rate of Progression to Simian-AIDS in Rhesus Macaques (Macaca mulatta)
Source: PLoS Genet. 2009 Jan 23;5(1):e1000346. doi: 10.1371/journal.pgen.1000346 (PMC2621346; doi:10.1371/journal.pgen.1000346)
Supplement: Table S4 — Microsatellite id, number of alleles found in the retrospective sample, and heterozygosity for the 53 typed microsatellites. (0.04 MB PDF) [file pgen.1000346.s011.pdf]

Table S4. Microsatellite id, number of alleles found in the retrospective sample, and heterozygosity for the 53 typed microsatellites.

MARKER	Number of alleles	Heterozygosity
D11S2002	7	0.76
D12S67	23	0.91
D13S280	14	0.87
D15S108	14	0.85
D17S1605	13	0.81
D18S1140	12	0.87
D18S1371	12	0.87
D1S231	12	0.85
D22S280	15	0.86
D2S296	22	0.91
D3S1768	13	0.88
D5S1989	12	0.86
D6S266	11	0.88
MML10S27	12	0.82
MML11S2	13	0.81
MML12S29	13	0.80
MML12S9	11	0.76
MML13S7	13	0.74
MML14S21	13	0.87
MML14S27	12	0.72
MML15S21	15	0.89
MML15S3	19	0.88
MML16S27	16	0.79
MML16S46	9	0.74
MML17S39	8	0.76
MML19S19	7	0.81
MML19S27	13	0.82
MML1S1	5	0.53
MML1S42	12	0.82
MML1S8	15	0.78
MML20S35	13	0.73
MML20S36	12	0.85
MML2S34	11	0.84
MML2S41	17	0.88
MML3S16	6	0.69
MML3S4	12	0.84
MML3S43	11	0.82
MML3S9	21	0.93
MML4S21	18	0.90
MML4S8	13	0.85
MML5S25	15	0.87
MML5S32	14	0.82
MML5S38	10	0.85
MML6S27	10	0.73
MML7S2	11	0.75
MML7S9	14	0.87
MML8S40	7	0.60
MML8S56	10	0.84
MML8S7	9	0.76
MML9S30	11	0.81
MML9S44	18	0.88
MML9S6	12	0.83
MMl1S6	12	0.71
