# Supplementary material for: Copy Number Variation of CCL3-like Genes Affects Rate of Progression to Simian-AIDS in Rhesus Macaques (Macaca mulatta)
Source: PLoS Genet. 2009 Jan 23;5(1):e1000346. doi: 10.1371/journal.pgen.1000346 (PMC2621346; doi:10.1371/journal.pgen.1000346)
Supplement: Text S1 — Additional methods describing validation of rtPCR primers and probes, analysis of microsatellite data, and power analysis. (0.05 MB DOC) [file pgen.1000346.s012.doc]

**Copy number variation of *CCL3-*like genes affects rate of progression to simian-AIDS in rhesus macaques (*Macaca mulata)***

**Supplementary Methods**

Confirming Primer sequences

Real-time PCR primer and probe sequences were designed against the publicly available rhesus macaque genome sequence. As the sequence is based on the genome of one Indian origin individual we tested the specificity of the primers by sub-cloning and sequencing PCR products from two Chinese and two Indian individuals. To do so, we designed primers that flank the original rtPCR primers, thereby amplifying a product that includes the entire original rtPCR product. We used TA-cloning to clone individual PCR products, and performed touch-down PCR followed by direct sequencing of 60 clones from each individual. The sequences were aligned and sequence differences called, using the Sequencher software (Gene Codes Corp. Ann Arbor, MI). While some polymorphisms were observed between the clones, there were no fixed differences observed between the groups (Table S2). Since most of the differences were observed in the Chinese origin individuals, the possible bias of the rtPCR assay is in the direction of underestimating *CCL3L* copy number of Chinese origin individuals, a conservative bias with respect to our conclusions.

Analysis of microsatellite data

In order to test for population structure and relatedness between individuals we typed 53 microsatellite loci (developed as part of the rhesus genome map at the Southwest National Primate Research Center; Rogers et al. 2006 <http://www.snprc.org/linkage/index.html>) in each of the 57 previously infected rhesus monkeys (see Table S4). PCR amplifications used 25 ng of genomic DNA as template, plus standard buffers, one unlabelled primer and one fluorescently labeled primer in reactions of 6 μl total volume. Thermocycling parameters differ among loci, but are available at http://www.snprc.org/linkage/index.html. Seven to ten PCR products were combined into single pools, an aliquot of LIZ-600 size standard (ABI) plus formamide was added, and this mixture loaded into the ABI 3730 instrument for capillary electrophoresis. Standard methods were used to determine each genotype for each individual sample. An image file of the raw data for multiplexed genotypes is created by installed ABI collection software. This image file was then analyzed using ABI GeneMapper software (Applied Biosystems Inc., Foster City, CA), employing the local Southern method and the internal ABI size standards to estimate fragment lengths. These results were used to create categories or “bins” of PCR fragments and to assign each individual band or allele to a single bin. Initial binning was done automatically using GeneMapper, each genotype was then checked by hand to ensure accuracy of the genotype calls. The levels of polymorphism, chromosomal locations, and allele size for these 57 loci were known previously (Rogers et al. 2006; <http://www.snprc.org/linkage/index.html>). This set of loci was selected for high heterozygosity and by avoiding pairs of closely linked markers, thus ensuring that genotypes are statistically independent across loci.

These data were used for three analyses. First, we conducted Structure (Pritchard et al 2000) and Principle Component Analysis (PCA) to ensure population-of-origin had been correctly assigned for animals in the retrospective samples. Second, we estimated pair-wise relatedness among individuals to detect cryptic relatedness in the samples and lastly, we conducted replicate association analyses as a form of genomic controls to assess the significance of the *CCL3L* association.

*Assessing population of origin*

To ensure the individuals in the retrospective sample were correctly attributed to their population of origin and to ensure none were of admixed heritage, we used Structure and PCA. Structure was run on the complete set of microsatellites for 500,000 iterations with a burn-in of 100,000 iterations. Three independent runs of *K* =2 were run using the admixture model with correlated allele frequencies and pop-flags off. Convergence of each run was evaluated by visual inspection of the Ln(P|D) plots. The results of the Structure runs confirmed that all individuals in the retrospective sample had been correctly assigned to their population. With the exception of two individuals, all showed q-values greater than 98%. These two individuals showed the greatest extent of admixture with q-values of ~94% (Figure S2). Additional confirmation of population assignment is provided by a principle component analysis (Figure S3A & S3B) conducted using the prcomp function in R. This analysis confirms that all samples have been correctly attributed to their population of origin.

*Cryptic relatedness*

As the individuals used in the retrospective analysis were all obtained from colonies, we used the microsatellite data to determine if there are significant levels of relatedness in the sample. Briefly, we used the modified Queller and Goodnight (QG; Queller and Goodnight 1989, Lynch and Ritland 1999) estimator of *r* to evaluate pair-wise relatedness of all individuals in the sample. We find that the Indian origin samples show, on average, higher levels of relatedness than the Chinese origin animals (Figure S4 A). This result is expected, as export of Indian-origin animals has been banned since 1978. Therefore, the Indian-origin animals have been isolated for a longer period time than the Chinese origin animals. Both populations show modest levels of cryptic relatedness (e.g., several pairs of individuals show QG distances between half sib relationships [0.25] and first cousins [0.0625] corresponding to dark black squares in Figure S6B & Figure S6C). This level of relatedness, however, appears to cause only a slight bias (if at all) in our estimates of the significance of the association; see below.

*Genomic control*

To further assess the significance of CNV in the *CCL3L* locus with time until onset, we conducted replicate association analysis with each microsatellite allele. For each allele at each locus we recoded the microsatellite as 2 (homozygous for the allele), 1 (heterozygous with the particular allele to be analyzed and another allele), and 0 (two alleles which are not that allele being analyzed). For each recoded allele we then conducted a Cox proportional hazard analysis with population origin as a covariate and recorded the *p*-value. We found that the distribution of the *p*-values closely follows a uniform distribution by visualizing the Q-Q plot (Figure S5A). The low level of relatedness seen in the above analysis may be causing the slight uptick in the distribution of the most extreme *p*-values. However, we find that the *p*-value for the association of *CCL3L* copy number with time until onset still falls in the extreme right tail of the *p*-value distribution (Figure S5B). Therefore, the relatedness seen in the above analysis is likely not significantly affecting the estimation of the association.

Power assessment and simulations for Chinese-origin Cox proportional Hazard model

In order to test whether the lack of an observed significant association between survivorship and *CCL3L* copy number for the Chinese-origin sample was due to power, we used a variant of non-parametric bootstrap re-sampling. In particular, we were interested in understanding whether the smaller sample size of the Chinese-origin sub-sample (*n* = 20), coupled with higher overall population mean *CCL3L* copy number accounted for the lack of a significant regression coefficient. In order to test this hypothesis, we generated *B* = 100 bootstrap data sets of size *n* = 20 or size *n* = 37 using the *Indian-origin* individuals sampled in proportion to the observed *CCL3L* copy number distribution in either the Chinese-origin sample (designated as “Chinese-like”) or the Indian-origin sample. For each data set, we sample with replacement triplets of (*CCL3L* copy number, time since infection, survivorship status) until either *n* = 20 or *n* = 37 individuals had been sampled. Each data set was then run through the same Cox proportional hazard regression analysis, and the *p*-value of the likelihood ratio test comparing models *m*_0_ (no factors) vs. *m*_1_ (*CCL3L* as a factor) were retained.

We observe that the distribution of *p*-values among replicates with “Chinese-like” *CCL3L* CNV distribution is markedly uniform in comparison to the *p*-value distribution for animals with “Indian-like” *CCL3L* CNV distribution. This indicates a much lower power in the former as compared to the latter. In other words, in the “Chinese-like” simulations we observe few data sets with significant *p*-values, where as in the “Indian-like” simulations upwards of 50% - 80% of simulations where in the lowest *p*-value bin. This result holds regardless of whether *n* = 20 or *n* = 37 individuals are sampled, although the power for *n* = 20 in the Indian bootstrap simulations is also reduced. Our interpretation of this finding is that the Chinese-only sample has little power to detect an effect of *CCL3L* copy number on survivorship due to a lack of animals with low number of copies of *CCL3L* and smaller overall sample size.

References:

Pritchard, J. K., Stephens, M., and Donnelly, P. Inference of population structure using multilocus genotype data. *Genetics* **155** 945-959 (2000).

Rogers, J., R. Garcia, W. Shelledy, J. Kaplan, A. Arya, Z. Johnson, M. Bergstrom, L., Novakowski, P. Nair, A. Vinson, D. Newman, G. Heckman and J. Cameron. An initial genetic linkage map of the rhesus monkey (*Macaca mulatta*) genome using human microsatellite loci. *Genomics* **87**, 30-38 (2006).

Queller D.C., K.F Goodnight. Estimating relatedness using genetic markers. *Evolution* **43,** 258-275 (1989).

Lynch M, Ritland K. Estimation of pairwise relatedness with molecular markers. *Genetics* **152,** 1753-1766 (1999).
